# Retinal non-perfusion progression in severe non-proliferative and proliferative diabetic retinopathy over time: INSPIRED study report 2

**DOI:** 10.1038/s41433-026-04707-7

**Published:** 2026-07-04

**Authors:** Wei-Shan Tsai, Sarega Gurudas, Hagar Khalid, Francesca Lamanna, Eleonora Riotto, Taffeta Ching Ning Yamaguchi, Louise Roch, Sobha Sivaprasad

**Affiliations:** 1 NIHR Moorfields Clinical Research Facility and Biomedical Research Centre, Moorfields Eye Hospital NHS Foundation Trust, 162 City Road, London, EC1V 2PD UK; 2 Hampshire Hospitals NHS Foundation Trust, Hampshire, UK; 3 Tanta University Faculty of Medicine, Tanta, Egypt; 4 Boehringer Ingelheim International GmbH, Birkendorfer Str. 65, 88397 Biberach, Germany

**Keywords:** Prognostic markers, Medical imaging

## Abstract

**Background/objectives:**

To determine the topographic distribution of retinal non-perfusion (RNP) progression in patients with severe non-proliferative diabetic retinopathy (sNPDR) and proliferative diabetic retinopathy (PDR).

**Subjects/methods:**

This retrospective study was conducted at Moorfields Eye Hospital in the United Kingdom. Patients with diabetic retinopathy (DR) who had two sets of ultra-widefield fundus fluorescein angiography were included. A grid comprising seven concentric rings was superimposed on the images. RNP areas were annotated using Optos’ proprietary tool. Total and zonal non-perfusion index (NPI) were calculated as the RNP area divided by the gradable retinal area. Adjusted linear mixed-effects models (LMEMs) were used to estimate progression, accounting for repeated measures and inter-eye correlation.

**Results:**

A total of 53 patients (67 eyes) were included in the analyses. This cohort comprised 19 (28%) eyes with PDR, 31 (46%) with sNPDR, and 17 (25%) with mild-to-moderate NPDR. Focusing on sNPDR and PDR, the nasal 20-30 mm zone had the largest NPI (0.37 ± 0.26) at baseline. Over time, the total NPI increased significantly, with an estimated change of +0.027 (12.42 mm²) per year (95% confidence interval [CI] = 0.008 to 0.045, *P* = 0.005). The inferior 10–20 mm ring showed the fastest NPI progression ( + 0.049/year; *P* = 0.007).

**Conclusions:**

This study demonstrates that NPI increased with time in DR. Notably, the inferior 10-20 mm zone was most vulnerable to NPI progression. Of note, this study was limited by the confounding effect of anti-vascular endothelial growth factor (anti-VEGF) treatment; therefore, a future prospective study to control for treatment exposure would be warranted.

## Introduction

It is estimated that a third of people with diabetes who undergo population-based retinal screening have diabetic retinopathy (DR), and one in ten cases of DR is vision-threatening [1, 2]. Retinal non-perfusion (RNP) is a major driver of DR progression. In 1984, Niki et al. first described the observation of RNP in non-proliferative DR (NPDR) eyes on fundus fluorescein angiography (FFA) [3]. By 1991, RNP was found by the Early Treatment Diabetic Retinopathy Study (ETDRS) to be a predictor of progression from NPDR to PDR [4].

With the advent of ultra-widefield FFA (UWF-FFA), RNP can now be quantified with greater accuracy. An established method for RNP measurement is the non-perfusion index (NPI), defined as the total measurable area of RNP divided by the total gradable retinal area [5]. Dr Silva et al. illustrated a significant rising trend of NPI with DR severity scale (DRSS) from 0.10 ± 0.13 (mild NPDR), 0.15 ± 0.17 (moderate NPDR), 0.19 ± 0.20 (severe NPDR) to 0.23 ± 0.20 (PDR) using Optos’ proprietary tool with DICOM Supplement 173 [6, 7]. Several studies have also demonstrated a positive correlation between NPI and the DRSS [3, 4, 6–10]. Notably, RNP of ≥100 disc area (DA) is associated with progression to PDR, with peripheral RNP having greater significance [11].

The RECOVERY study used proprietary software that combined the Optos optical model with the Navarro model eye to assess total NPI in 40 PDR eyes without diabetic macular oedema (DMO), yielding a similar value of 0.258 ± 0.153 [9]. However, the DAVE study, which evaluated 40 eyes with DMO and severe NPDR, reported a slightly higher NPI of 0.31 ± 0.19 [8]. These observations suggest that total NPI may not be sufficient to predict progression to PDR, and the location of RNP may play a crucial role in determining the rate of DR progression to PDR [6, 7, 12].

Numerous studies have examined the prognostic impact of RNP location [3, 7, 8, 13–16]. Initially, Niki et al. reported an ascending order of DR progression in peripheral, mid-peripheral, central, and generalised RNP subtypes [3]. Later, Antaki et al. showed that both the central non-perfusion (CNP) index and peripheral non-perfusion (PNP) index increased in PDR [16]. Interestingly, Ishibazawa et al. observed greater RNP in the area adjacent to arteries than near veins [13].

Most studies have now adopted the protocol AA three-concentric-ring method to examine NPI [6, 17]. If measured in diameter, 20 mm, 30 mm, and >30 mm each represent the posterior pole, mid-periphery, and far-periphery, respectively. Using this method, the DAVE study illustrated an increasing trend in NPI from 0.22 (posterior pole) to 0.37 (mid-periphery) and 0.43 (far periphery) [8]. Their subset study reported that the inferonasal quadrant had the highest NPI (0.43 ± 0.3) [14]. Most importantly, Dr Silva et al. proposed that eyes with higher NPI at the posterior pole and mid-periphery had a higher risk of DR worsening at 4 years [12].

To date, measuring changes in RNP over time remains challenging. The total gradable retina in UWF-FFA may vary between visits due to artefacts, the timing of the FFA sequence, and the total gradable retina. Therefore, a location within a specified grid that is present at baseline and at follow-up visits is required to determine the actual change in RNP.

This study aimed to assess the topographic distribution of RNP within a pre-specified grid divided into zones. We then evaluated the changes in RNP using the same pre-specified grid. Locations most susceptible to DR progression with time were identified, and their associated risk factors were studied. These results will provide valuable information for DR clinical trials, such as the MAGIC study (NCT05681884) and the CRIMSON study (NCT06321302), which have used various RNP measures as outcomes [18–20].

## Methods

### Study design

This study, entitled **I**nvestigation of Retinal **N**on-Perfu**s**ion and Visual Function in **P**roliferat**i**ve and Non-Prolife**r**ative Retinopathy in Patients with **D**iabetes (**INSPIRED**), was a retrospective chart review (audit registration number 1200) based at the Moorfields Clinical Research Facility. The study followed the principles of the Declaration of Helsinki.

### Patients

Patients with any level of DR who underwent Optos UWF-FFA at two visits between 2010 and 2024 were identified. The exclusion criteria were prior panretinal photocoagulation (PRP), media opacity (e.g., vitreous haemorrhage or cataract), poor image quality, and other ischaemic retinopathies (e.g., retinal vein occlusion). Their demographic data, medical and ocular history, and best recorded visual acuity (BRVA) were collected from electronic medical records. Their colour fundus photo (CFP) and FFA were obtained from the hospital’s Optos patient database (Optos P200DTx, Dunfermline, Scotland, UK).

### Image processing

**Fig. 1:**
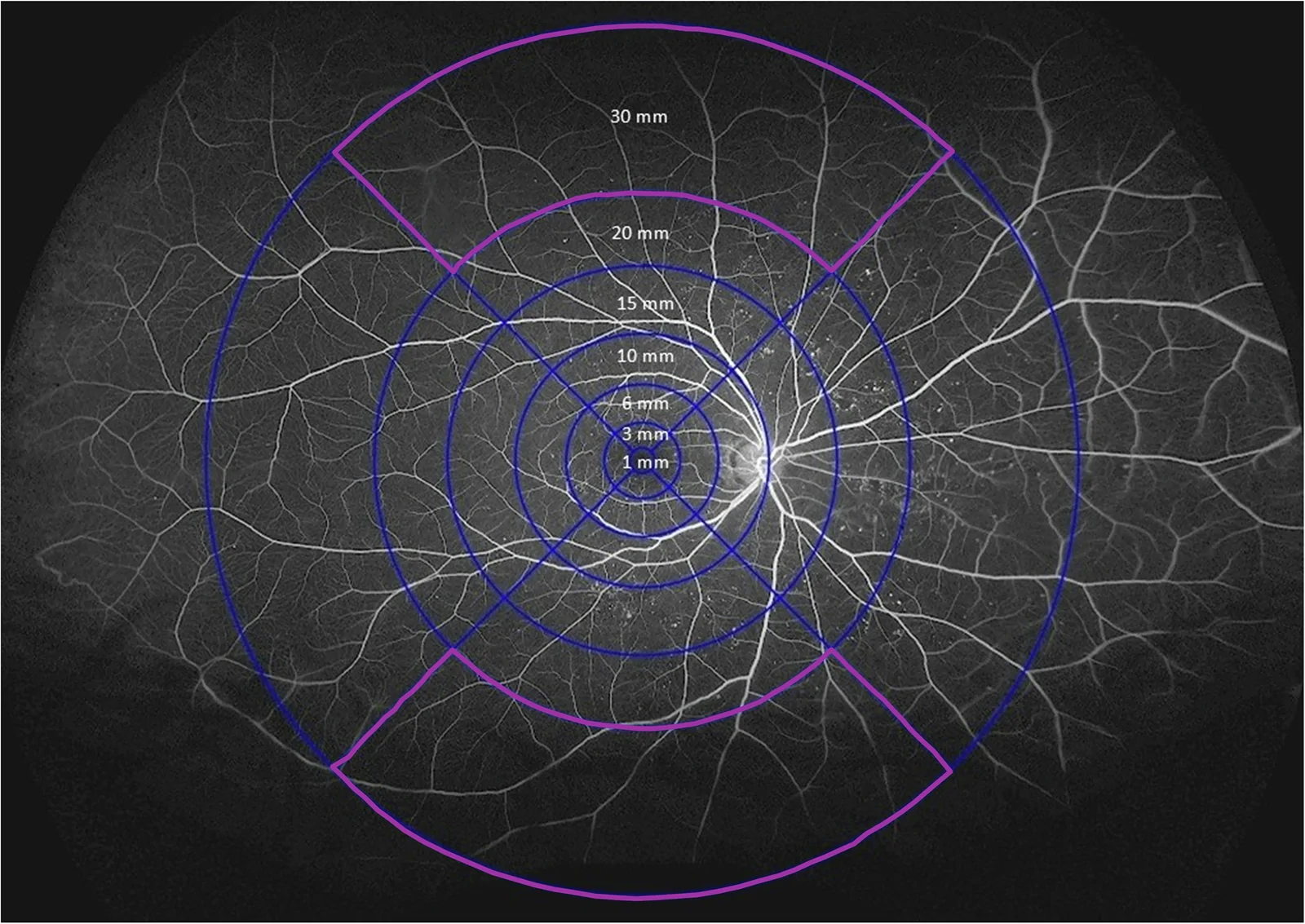
A demonstration of the INSPIRED study grid. This specially designed grid features seven concentric rings with diameters of 1, 3, 6, 10, 15, 20, and 30 mm. From the 3-mm ring onwards, each ring is divided into four quadrants, giving 25 study subfields. Notably, compared with protocol AA, the 0–20 mm zone corresponds to the posterior pole, the 20–30 mm zone to the mid-periphery, and the area beyond 30 mm to the far periphery. In the present study, the superior and inferior 20–30 mm rings (highlighted in purple) were not annotated because they were considered significantly affected by eyelash artefact.

Once deemed eligible, the Optos images were copied and saved to a separate study folder using an anonymised study identifier. On the Optos viewing platform, a specially designed “INSPIRED grid” composed of 7 concentric rings with diameters of 1, 3, 6, 10, 15, 20, and 30 mm was overlaid onto the images and centred at the macula (Fig. 1). The 1-, 3-, and 6-mm rings were designed to match the ETDRS ring on optical coherence tomography (OCT) and represent macula RNP. From the 3-mm ring onwards, each ring is divided into four quadrants, giving 25 study subfields. Notably, the 0 to 20 mm diameter zone equals the posterior pole, the 20 to 30 mm diameter zone equals the mid-periphery, and the area beyond the 30 mm diameter zone equals the far periphery.

All measurements were performed using Optos’ proprietary tool, which incorporates DICOM Supplement 173 [21]. In brief, this tool automatically corrects measurements using spherical trigonometry, thereby accounting for the disparity between two-dimensional computer screen images and the actual retinal areas in human eyes. The precision and accuracy of the tool’s measurement have been previously reported [22].

### Diabetic retinopathy grading

The entire gradable UWF CFP was used for grading DR characteristics (Global DRSS). We graded the presence and location of microaneurysms (MAs), retinal haemorrhage (RH), venous beading (VB), intra-retinal microvascular abnormalities (IRMA), neovascularisation of the disc (NVD) and elsewhere (NVE). Using the above results, four highly trained retinal specialists (WT, HK, FL, ER) determined the global DRSS according to the ETDRS classification (Supplementary Table 1) [23, 24]. In addition, the presence of predominantly peripheral lesions (PPL) was also evaluated according to the definition of more than 50% of DR characteristics outside of the traditional ETDRS 7-field grid [12]. The same PPL evaluation was also applied to FFA scans. The graders attended weekly consensus meetings, and any discrepancies were adjudicated by a senior retinal consultant (SS). The final inter-grader agreement for global DRSS and PPL was excellent ( > 75%) [25].

### Retinal non-perfusion determination

The graders carefully selected a single FFA scan from the arteriovenous phase with the highest image quality for grading. We utilised the maximum “enhance” function to increase capillary resolution and adjusted the “gamma” value to an appropriate brightness, allowing RNP to be differentiated from the perfused retina. The RNP area was defined as the area with the same grayscale as the foveal avascular zone. Alternatively, the loss of 4^th^-order capillaries relative to the neighbouring retina was included [9]. The Optos proprietary tool automatically calculates the pixel count within an area of interest and converts it to square millimetres (mm²). The minimal required area for defining RNP was 0.127 mm² [26]. The same clinician graded the FFA scans at two visits. We did not annotate the superior and inferior 20-30 mm subfields, as most of them were rendered ungradable by eyelashes. The RNP area was summed in each subfield, and the total non-perfusion area (NPA) readout was the average of two matched graders. The non-perfusion index (NPI) was calculated as the NPA divided by the gradable area in that subfield. The gradable area of each subfield was reported in our previous publication [25].

### Statistical analysis

For continuous variables, the results were presented as means and standard deviations. For categorical variables, results were presented both numerically and as percentages. Spearman’s rho and Kendall’s tau correlation coefficients were reported to assess the correlation between NPI total and in different rings with DRSS grading, with clustered bootstrap 95% confidence intervals based on 5,000 replicates. Confidence intervals account for within-subject clustering (bilateral eligibility in some participants) and the clustered bootstrap was computed using the *ClusterBootstrap* package in R [27]. To examine changes in the relevant NPI metric over time, we fitted linear mixed-effects models (LMEMs) with the NPI metric as the dependent variable. Each model included a random intercept for participant ID to account for within-participant correlations arising from repeated measures at visits 1 and 2 and from the inclusion of data from both eyes in some patients. Time was modelled as a continuous fixed effect to accommodate varying follow-up durations, and the resulting coefficient represents the estimated yearly change in the NPI metric. Models were additionally adjusted for baseline age, sex, DRSS, and BRVA. Analyses of change over time were restricted to eyes with severe NPDR and PDR to reduce heterogeneity in disease stage. Earlier stages of diabetic retinopathy (mild/moderate NPDR) may exhibit slower and potentially non-linear progression, with periods of relative stability followed by worsening, which may not be well captured given that only two FFAs (baseline and follow-up) were available in the analysis cohort. To assess whether the growth of total NPI differs between severe NPDR and PDR eyes, we fitted separate LMEMs with total NPI as the dependent variable, again including a random intercept for participant ID to account for within-participant correlations from repeated measures and bilateral data. Fixed effects included the main effects of baseline DR severity and its interaction with time. The reported statistics represent estimated time slopes, corresponding to the yearly change in total NPI within each predictor category. For the reference category of each predictor, the slope reflects the main effect of time; for all other categories, the slope equals the sum of the main effect of time and the relevant interaction term. Category-specific slopes and associated *P*-values were obtained directly from the fitted models using the *emmeans* package, with the Kenward–Roger method to estimate denominator degrees of freedom.

## Results

A total of 53 patients (67 eyes) were eligible for this study. At baseline (Table 1 and 2), the mean age of the cohort was 56 ± 13 years, with a slight majority of male candidates (55%). The average BRVA was 0.14 ± 0.18 logMAR. Most eyes had severe NPDR (DRSS level 53) (46%; *N* = 31), followed by 28% (*N* = 19) PDR (DRSS level ≥61) and 25% (*N* = 17) mild to moderate NPDR (DRSS level 20-47). Of the 67 eyes, the proportion with CFP PPL and FFA PPL was 18% (*N* = 12) and 21% (*N* = 14), respectively. Notably, 9 eyes (13%) had prior anti-vascular endothelial growth factor (anti-VEGF) injection, and 10 eyes (15%) declined or missed their PRP treatment. At baseline, the total NPI also exhibited a moderate correlation with the DRSS (ρ = 0.50) (Supplementary Fig. 1). A similar correlation was also observed between the 10–20 mm ring and the DRSS.

**Table 1:** Summary of demographics at baseline.

Demographics	All patients (*n* = 53)
Age, mean (SD), years	56 (13)
<50 years old, No. (%)	16 (30%)
50-60 years old, No. (%)	13 (25%)
>60 years old, No. (%)	24 (45%)
Sex	
Male, No. (%)	29 (55%)
Female, No. (%)	24 (45%)
Ethnicity	
Asian, No. (%)	12 (33%)
Black, No. (%)	4 (11%)
White, No. (%)	13 (36%)
Other, No.	7 (19%)
Not stated	17
Bilateral recruitment, No. (%)	14 (26.4%)

*SD* standard deviation.

**Table 2:** Summary of ocular characteristics at baseline.

Characteristics	All eligible eyes (*n* = 67)
BRVA (logMAR), mean (SD); median [Q1, Q3]	0.14 (0.18); 0.10 [0.00, 0.18]
<0.3 logMAR, No. (%)	48 (72%)
≥0.3, <1.0 logMAR, No. (%)	7 (10%)
≥1.0 logMAR	12 (18%)
Lens pseudophakia, No. (%)*	18 (28%)
DR severity	
Mild NPDR (DRSS 20-35), No. (%)	8 (12%)
Moderate NPDR (DRSS 43-47), No. (%)	9 (13%)
Severe NPDR (DRSS 53), No. (%)	31 (46%)
PDR (DRSS 61 above), No. (%)	19 (28%)
History of IVI, No. (%)	9 (13%)
History of macular laser, No. (%)*	7 (11%)
History of PPV, No. (%)*	1 (1.5%)
CFP PPL presence, No. (%)	12 (18%)
FFA PPL presence, No. (%)	14 (21%)

*BRVA* best-recorded visual acuity, *CFP* colour fundus photo, *DR* diabetic retinopathy, *DRSS* diabetic retinoapthy severity scale, *FFA* fundus fluorescein angiography, *IVI* intravitreal injection, *logMAR* logarithm of the Minimum Angle of Resolution, *NPDR* non-proliferative diabetic retinopathy, *PDR* proliferative diabetic retinopathy, *PPL* predominant peripheral lesion, *PPV* pars plana vitrectomy.

* No record = 3 for lens status, 1 for macular laser.

We then focused on analysing eyes with sNPDR and PDR at baseline that had a second FFA within 30 months (*N* = 48 eyes). Table 3 showed that these eyes exhibited a yearly progression rate of +0.027/year, with a 95% CI of 0.008 to 0.045 (*P* = 0.005) following adjustment for baseline age, sex, DRSS score and BRVA. Among all sectors, the inferior 10–20 mm ring showed the fastest NPI expansion potential ( + 0.049/year, 95% CI, 0.014 to 0.083; *P* = 0.007). Eyes with sNPDR at baseline showed a greater increase in total NPI over time ( + 0.037/year; *P* < 0.001, Supplementary Table 2). In contrast, eyes with PDR showed no evidence of change in total NPI (-0.003/year; *P* = 0.86).

**Table 3:** NPI Progression in Severe NPDR and PDR with a Follow-Up Time ≤30 Months.

				Unadjusted		Adjusted^b^	
*N* = 48 eyes^a^	First visit mean (SD)	Second visit mean (SD)	Raw yearly change, change per 1 yearly increase Mean (SD)	LMEM^c^: change per 1 year increase in time, beta (95% CI)	*P*-value	LMEM^bc^: change per 1 year increase in time, beta (95% CI)	*P*-value
Total NPI	0.22 (0.16)	0.24 (0.15)	0.020 (0.079)	0.027 (0.008 to 0.045)	0.005	0.027 (0.008 to 0.045)	0.005
Central NPI	0.58 (0.18)	0.61 (0.16)	0.017 (0.302)	0.029 (-0.006 to 0.064)	0.11	0.027 (-0.008 to 0.062)	0.13
Nasal NPI	0.26 (0.17)	0.28 (0.17)	0.016 (0.080)	0.024 (0.006 to 0.042)	0.01	0.023 (0.005 to 0.041)	0.01
1-10 mm ring	0.04 (0.07)	0.03 (0.05)	-0.013 (0.053)	-0.003 (-0.015 to 0.008)	0.57	-0.003 (-0.015 to 0.009)	0.59
10-20 mm ring	0.17 (0.13)	0.19 (0.15)	0.004 (0.087)	0.026 (0.006 to 0.047)	0.01	0.026 (0.006 to 0.047)	0.01
20-30 mm ring	0.37 (0.26)	0.40 (0.26)	0.032 (0.128)	0.030 (0.001 to 0.059)	0.04	0.029 (0.000 to 0.058)	0.047
Superior NPI	0.19 (0.17)	0.21 (0.17)	0.020 (0.105)	0.023 (0.001 to 0.045)	0.04	0.024 (0.002 to 0.046)	0.03
1-10 mm ring	0.09 (0.10)	0.08 (0.10)	-0.005 (0.092)	-0.004 (-0.022 to 0.014)	0.64	-0.005 (-0.023 to 0.013)	0.60
10-20 mm ring	0.22 (0.21)	0.25 (0.21)	0.029 (0.126)	0.033 (0.006 to 0.060)	0.02	0.034 (0.007 to 0.061)	0.02
Temporal NPI	0.21 (0.20)	0.24 (0.20)	0.017 (0.136)	0.026 (0.000 to 0.052)	0.049	0.026 (-0.000 to 0.052)	0.05
1-10 mm ring	0.12 (0.14)	0.10 (0.11)	-0.007 (0.088)	-0.011 (-0.032 to 0.010)	0.28	-0.012 (-0.033 to 0.009)	0.26
10-20 mm ring	0.12 (0.16)	0.14 (0.16)	0.010 (0.123)	0.023 (-0.004 to 0.051)	0.09	0.023 (-0.004 to 0.051)	0.10
20-30 mm ring	0.29 (0.29)	0.34 (0.30)	0.027 (0.200)	0.037 (0.002 to 0.073)	0.04	0.037 (0.002 to 0.073)	0.04
Inferior NPI	0.17 (0.14)	0.22 (0.17)	0.031 (0.122)	0.040 (0.012 to 0.068)	0.006	0.038 (0.010 to 0.066)	0.008
1-10 mm ring	0.08 (0.10)	0.09 (0.12)	0.008 (0.111)	0.008 (-0.013 to 0.029)	0.45	0.008 (-0.013 to 0.029)	0.44
10-20 mm ring	0.21 (0.17)	0.26 (0.21)	0.039 (0.155)	0.051 (0.016 to 0.086)	0.005	0.049 (0.014 to 0.083)	0.007

*CI* confidence interval, *LMEM* linear mixed-effects models, *NPDR* non-proliferative diabetic retinopathy, *NPI* non-perfusion index, *PDR* proliferative diabetic retinopathy, *SD* standard deviation.

^a^Two eyes with follow-up >30 months were removed due to potential influence on model estimates.

^b^Adjusted for age, sex, baseline BRVA and DRSS score.

^c^Separate linear mixed-effects models were fitted for each outcome to account for within-participant correlation due to repeated measures at baseline and follow-up, and eyes nested within participant. Time was included as a continuous predictor, and the corresponding beta coefficients for time are reported. The dependent variables are listed in the leftmost column.

## Discussion

This study revealed several characteristics of RNP in a cohort predominantly composed of middle-aged Asian and White people with severe NPDR to PDR. Firstly, DRSS showed a positive correlation with the NPI, with the strongest correlation observed in the total and 10–20 mm rings, suggesting the important role of global and posterior pole RNP in DR progression. Secondly, we estimated the NPI progression rate among sNPDR/PDR cases would be +0.027/year, which is even faster in the inferior 10–20 mm ring ( + 0.049/year).

The concept of increasing NPI with DR severity is not novel. Our study revealed a moderate correlation between total NPI and DRSS in a cohort comprising mild NPDR (12%), moderate NPDR (13%), severe NPDR (46%), and PDR (28%). This phenomenon has been previously demonstrated by Dr Silva et al. Specifically, their team noted a twofold difference in NPI between eyes without DR and those with PDR [7]. Interestingly, NPI typically stabilises during the PDR stage, ranging from 0.08 to 0.34 to 0.50 in mild, moderate, and severe NPDR, respectively [28]. However, the NPI plateaued at 0.48 and 0.50 for low-risk and high-risk PDR, respectively [28], which might explain the moderate, rather than strong, correlation between total NPI and DRSS. Our supplementary Table 2 also suggested that NPI progressed more quickly in sNPDR than PDR.

The location of RNP has recently attracted researchers’ attention. Our study identified that the nasal mid-periphery exhibited the largest NPI (0.37 ± 0.26) in sNPDR/PDR eyes. This finding corroborates DAVE’s subset study, where they found the infero-nasal quadrant had the largest NPI (0.43 ± 0.30) [14]. However, in a heat map generated by Dr Silva et al., the superior and temporal periphery showed a greater predilection for RNP [7]. In addition, they found that NPI at the posterior pole and mid-periphery was associated with a poor prognosis for DR at 4 years [12]. Our study suggests the inferior 10-20 mm NPI progressed the quickest among all sectors in sNPDR/PDR. Nevertheless, all these studies were conducted on FFA scans; therefore, the discrepancy may be attributable to fluorescein leakage and scattering, eyelash artefacts, or the presence of DMO and diabetic macular ischaemia (DMI). These differences may also suggest novel RNP-related phenotypes that warrant further investigation.

Currently, only one study from the Northwestern University team has formally examined the NPI progression rate over time in DR, which was estimated at 0.45 ± 0.90% over two years, with greater progression in moderate and severe NPDR [29]. The scant evidence, understandably, can be attributed to challenges in conducting studies on NPI progression, including concerns about the risk of fluorescein anaphylaxis, inherent artefacts associated with FFA, a lack of consensus on RNP annotations, and difficulties in tracking RNP changes [17, 28]. Our present study suggests that the total NPI increases by 0.027/year (95% CI, 0.008 to 0.045), corresponding to a 12.42 mm²/year increase in sNPDR/PDR cases.

Regarding DMI, our previous natural history study using optical coherence tomography angiography (OCTA) demonstrated a consistent enlargement of the foveal avascular zone area in stable-treated PDR over one year (0.67 ± 0.39 mm² to 0.71 ± 0.44 mm², with an average rate of +0.04 mm²/year) [30]. This is very similar to the results of the present FFA-based study. However, the RISE and RIDE studies revealed that, without treatment, macular RNP increased significantly from 0.17 disc area (DA) to 0.27 DA at 24 months in the sham group (equivalent to +0.125 mm²/year) [31]. The higher progression rate observed in the RISE and RIDE studies is due to their inclusion of a circle up to 6 mm in diameter as part of the macula definition.

This study has several strengths. To begin, we applied our specially designed INSPIRED grid, which enables a more detailed description of the RNP location across four quadrants. It reveals more topographic detail than the traditional 3-zone method. Moreover, we can correlate the NPI in the central 1-, 3-, and 6-mm rings with the ETDRS grid on OCT images, if needed. Furthermore, we asked the same grader to conduct the quantitative evaluation of RNP progression after consensus training, thereby reducing intergrader variation.

We also acknowledge the limitations of the present study. First, given the retrospective nature of the study, we could not obtain the best-corrected visual acuity, nor could we control the treatments or the follow-up intervals. In addition, patients undergoing FFA likely had higher DRSS, and therefore, selection bias in this retrospective study is difficult to avoid. Our estimates are more applicable to real-world practice with variable anti-VEGF use rather than to treatment-naive populations, and actual progression rates in untreated cohorts may be higher. The rates we reported reflect treatment-modified progression rather than purely natural progression. Second, we did not study the RNP beyond the 30 mm zone, and we were unable to grade beyond 20 mm in the superior and inferior regions due to difficulties maintaining image quality, which might contribute to more accurate analytic results in the nasal and temporal quadrants. Third, the small sample size, as few people currently have two available FFA sets, may reduce the power of our findings. Finally, the scattering effect from FFA fluorescein may affect the precision of RNP measurement. We therefore recommend using wide-angle OCTA with a diameter of at least 30 mm to accurately estimate RNP changes over time in prospective settings.

## Conclusion

To conclude, our cohort has contributed new insights to RNP studies in DR by introducing a yearly change in RNP as the unit of measure in clinical trials. The most severe NPI progression was observed in the inferior 10–20 mm ring among sNPDR/PDR cases; therefore, it would be reasonable to target this region in future trial design.

## Summary

### What was known before

- Non-perfusion index (NPI) can be used to measure change in retinal non-perfusion (RNP).
- NPI progressed over time, and RNP at the posterior pole and mid-periphery had a higher risk of diabetic retinopathy (DR) progression.

### What this study adds

- Over time, the total NPI increased significantly, with an estimated change of +0.03 (12.42 mm²) per year in severe non-proliferative and proliferative DR.
- The inferior 10–20 mm ring showed the fastest NPI progression +0.05 per year.

## Supplementary information

Supplementary material

## Data Availability

The data collected for the current study, including individual patient data and a data dictionary that defines each field in the dataset, will not be made available to others. Please contact the corresponding author, Professor Sobha Sivaprasad.

## **Meeting presentations**

The results were partially presented at EURETINA 2025 in Paris.
