# Supplementary material for: Retinal non-perfusion progression in severe non-proliferative and proliferative diabetic retinopathy over time: INSPIRED study report 2
Source: Eye (Lond). 2026 Jul 4;40(14):2193–8. doi: 10.1038/s41433-026-04707-7 (PMC13601625; doi:10.1038/s41433-026-04707-7)
Supplement: Supplementary file 1 [file 41433_2026_4707_MOESM1_ESM.docx]

**Supplementary information**

**Supplementary Table 1.** Diabetic Retinopathy Severity Scale.

| **Severity** | **Level** | **Grading** | **Characteristics** |
| --- | --- | --- | --- |
| No DR | 1 | 10 | No DR characteristics |
| Very mild NPDR | 2 | 20 | MAs only |
| Mild NPDR | 3 | 35 | MAs ± mild RH ± hard exudates ± CWS |
| Moderate NPDR | 4 | 43A | Moderate RHs in 4 quadrants or severe in 1 quadrant. |
|  | 4 | 43B | Mild IRMA in 1-3 quadrants |
| Moderately severe NPDR | 5 | 47A | 43A + 43B |
|  | 5 | 47B | Mild IRMA in 4 quadrants |
|  | 5 | 47C | Severe RH in 2-3 quadrants |
|  | 5 | 47D | VB in 1 quadrant |
| Severe NPDR | 6 | 53A | ≥2 grade 47 characteristics |
|  | 6 | 53B | Severe RH in 4 quadrants |
|  | 6 | 53C | Moderate to severe IRMA in ≥1 quadrant |
|  | 6 | 53D | VB in ≥2 quadrants |
|  | 6 | 53E | ≥2 grade 53 characteristics |
| Mild PDR | 7 | 61 | NVE <1/2 DA in ≥1 quadrants |
| Moderate PDR | 8 | 65A | NVE ≥1/2 DA in ≥1 quadrants |
|  | 8 | 65B | NVD <1/4 to 1/3 DA |
| High-risk PDR | 9 | 71A | NVD ≥1/4 to 1/3 DA without VH or PRH |
|  | 9 | 71B | VH or PRH obscuring ≥1 DA |
| Very high-risk PDR | 10 | 75 | NVD ≥1/4 to 1/3 DA with VH or PRH |
| Advanced PDR | 11 | 81 | Retina obscured by VH or PRH |
| Very advanced PDR | 12 | 85 | VH or TRD at macula |
|  |  | 90 | Ungradable |

Abbreviations: CWS = cotton-wool spot; DA = disc area; DR = diabetic retinopathy; DRSS = Diabetic Retinopathy Severity Scale; IRMA = intra-retinal microvascular abnormality; MA = microaneurysm; NPDR = non-proliferative diabetic retinopathy; NVD = neovascularisation of disc; NVE = neovascularisation of elsewhere; PDR = proliferative diabetic retinopathy; PRH = pre-retinal haemorrhage; RH = retinal haemorrhage; TRD = tractional retinal detachment; VB = venous beading; VH = vitreous haemorrhage.

**Supplementary Figure 1**. Non-perfusion index (NPI) versus diabetic retinopathy severity scale (DRSS) grading across rings. Spearman’s rho and Kendall’s tau correlation coefficients are shown for each ring, with clustered bootstrap 95% confidence intervals based on 5,000 replicates. Confidence intervals account for within-subject clustering (bilateral eligibility in some participants). The total NPI presented with a moderate correlation (rho = 0.5) with the DRSS. The NPI in the 10-20 mm ring also had a similar relationship with the DRSS.

**Supplementary Table 2.** Association between Change in Total Non-Perfusion Index at Second Visit from Baseline Stratified by sNPDR and PDR eyes, with Follow-Up time ≤30 months.

| **N = 48 eyes^a^** | **First visit**  **mean (SD)** | **Second visit**  **mean (SD)** | **Raw yearly change, change per 1 year increase**  **Mean (SD)** | **LMEM^b^: change per 1 year increase in time, beta (95% CI)** | ***P*-value** |
| --- | --- | --- | --- | --- | --- |
| DRSS |  |  |  |  |  |
| Severe | 0.17 (0.13) | 0.22 (0.14) | 0.036 (0.066) | 0.037 (0.016 to 0.058) | <0.001 |
| PDR | 0.29 (0.17) | 0.28 (0.16) | -0.006 (0.091) | -0.003 (-0.042 to 0.035) | 0.86 |

Abbreviations: BRVA = best-recorded visual acuity; CI = confidence interval; DRSS = diabetic retinopathy severity scale; LMEM = Linear Mixed Effects Models; logMAR = logarithm of the Minimum Angle of Resolution; PDR = proliferative diabetic retinopathy; SD = standard deviation; sNPDR = severe non-proliferative diabetic retinopathy.

^a^ Two eyes with follow-up >30 months were removed due to potential influence on model estimates.

^b^ Linear mixed-effects models accounted for within-participant correlation arising from the inclusion of both eyes from the same individual and from repeated measures at visits 1 and 2. The outcome was total NPI. Fixed effects included the main effects of DR severity and its interaction with time. The reported statistics represent slope estimates for time, corresponding to the estimated annual change in total NPI within each DR severity category. Category-specific slopes and associated p-values were obtained directly from the fitted model using the emmeans package with the Kenward–Roger method for denominator degrees of freedom.
